# Neurofilaments support the differentiation of amyotrophic lateral sclerosis from degenerative cervical myelopathy

**DOI:** 10.1007/s00702-026-03277-y

**Published:** 2026-10-09

**Authors:** Julia Goebell, Franziska Bachhuber, Jutta Karmann, Veronika Klose, Jan Kassubek, Hans-Peter Müller, Hayrettin Tumani, Albert Ludolph

**Affiliations:** Department of Neurology, Institute for Applied Physiology, University of Ulm, Ulm, Germany

**Keywords:** Myelopathy, ALS, NfL pNfH, Biomarkers

## Abstract

The differential diagnosis between degenerative cervical myelopathy (DCM) and early amyotrophic lateral sclerosis (ALS) is often clinically challenging; laboratory tests that support this distinction could be highly valuable. This study examined the concentrations of neurofilament light chain (NfL) and the phosphorylated neurofilament heavy chain (pNfH), in CSF and serum of carefully selected patients with DCM, spinal canal stenosis (SCS) and ALS, after exclusion of major neurological confounders. Patients were assigned to five groups according to MRI findings and ALS diagnosis. All patients were treated in the Department of Neurology, and were only included if a CSF or serum sample was available for retrospective measurement of neurofilaments. Z-scores were used for age-adjusted interpretation of NfL concentrations in CSF and serum. In addition, the albumin quotient (QAlb), as a marker for the integrity of the blood-CSF barrier, was investigated in the five patient groups. The levels of NfL and pNfH, including Z-score distributions, differed significantly. The groups with diagnosed ALS had elevated neurofilaments both in CSF and Serum. Comparison of neurofilament concentrations between patients with isolated spinal canal stenosis and patients with cervical myelopathy revealed no significant difference. The QAlb showed no clear correlation with elevated neurofilaments and provided limited value for differential diagnosis. In summary, we showed that NfL and pNfH in CSF and serum support the differential diagnosis of cervical SCS with and without myelopathy from ALS in carefully selected cohorts. However, since neurofilaments are not specific, they should be used in context with clinical assessment and imaging for individual diagnostic and treatment decisions.

## Introduction

Degenerative cervical myelopathy (DCM) is the most common cause of cervical spinal cord damage (Nouri et al. 2020) and is characterised by chronic or subacute compression of the cervical spinal cord, resulting from degenerative changes which cause narrowing of the spinal canal and lead to progressive neurological dysfunction (Hejrati et al. 2023; Karadimas et al. 2015). Spinal canal stenosis (SCS) can be diagnosed using imaging techniques such as magnetic resonance imaging (MRI) or computed tomography (CT) (Hesni et al. 2023). Magnetic resonance imaging (MRI) is the standard imaging method used in diagnostics (Hesni et al. 2023; Nouri et al. 2016). MRI findings of spinal cord compression, in combination with increased T2 intraparenchymal signal abnormalities can help in the diagnosis of DCM (Harrop et al. 2010). In the T2-weighted sequence, intramedullary signal hyperintensity can be interpreted as a ‘myelopathy signal’ showing increased water content (Hesni et al. 2023).

The resulting impairment to the ascending and descending neuronal pathways is accompanied by corresponding signs of neurological dysfunction including radicular deficits in the upper extremities (Jiang et al. 2023). DCM presents with variable symptoms and progression, most patients undergo gradual clinical worsening, significantly affecting quality of life (Edwards et al. 2003; King et al. 2003). Multimorbidity, the variability of the clinical presentation and disease progression of patients with DCM make the differentiation from other diseases difficult and early diagnosis challenging (Hilton et al. 2022; Jiang et al. 2023).

The Japanese Orthopaedic Association (JOA) scoring system is used internationally to classify clinical findings. It takes into account motor function of the upper and lower extremities, sensory deficits and sphincter function (Edwards et al. 2003). The modified JOA (mJOA) criteria use a more differentiated scoring system which can help to classify the severity of myelopathy, support decisions on treatment and assess prognosis (Hilton et al. 2022; Tetreault et al. 2013, 2017).

Amyotrophic lateral sclerosis (ALS) represents a clinically significant differential diagnosis to cervical myelopathy, as its clinical presentation can be similar (Kassubek et al. 2012; Masrori and Damme 2020). Especially early-stage ALS symptoms can resemble those caused by radiculopathy, which makes the differentiation with the help of potential clinical biomarkers of major interest (Ariaei et al. 2025). ALS is a neurodegenerative disease of the first and second motor neuron and its average survival time from the onset of the first symptoms is approximately 2 to 5 years (Vasta et al. 2025). The ALS Functional Rating Scale-Revised (ALSFRS-R) is a validated clinical instrument used to measure functional impairment in ALS patients (Cedarbaum et al. 1999). It is used to assess disease progression in clinical practice and clinical trials (Kimura et al. 2006; Kollewe et al. 2008; Labra et al. 2016; Ludolph et al. 2024; Eijk et al. 2019). Like DCM, the clinical presentation of ALS is variable, with symptoms differing based on the site of disease onset and the pattern of motor neuron involvement (Masrori and Damme 2020).

Neurofilaments, a group of structural proteins from neurons which can be released into the CSF and blood following neuroaxonal damage, are promising biomarkers for the diagnosis of ALS. The light chain (NfL) and phosphorylated heavy chain (pNfH) of neurofilaments can be measured in CSF and blood. It has been shown that significantly elevated levels of neurofilaments are present in both CSF and serum in most ALS patients (Halbgebauer et al. 2022). Neurofilament concentration increases early in the course of ALS and remain at high levels (Feneberg et al. 2018; Steinacker et al. 2016). In serum, neurofilament concentrations are about ten times lower than in CSF, yet their levels in both blood and CSF correlate and reflect the disease progression rate of ALS (Halbgebauer et al. 2022). It has been reported that in DCM elevated neurofilament levels may indicate the severity of myelopathy as spinal cord compression progresses (Takahashi et al. 2018).

The aim of the current study was to find out whether NfL and pNFH in CSF and Serum can serve as diagnostic markers for DCM and SCS and whether these biomarkers may assist in the differential diagnosis against ALS. In an elderly patient group, as it is the case with DCM, we prioritized unequivocal diagnosis, rather than including large groups with numerous comorbidities.

## Methods

**Fig. 1:**
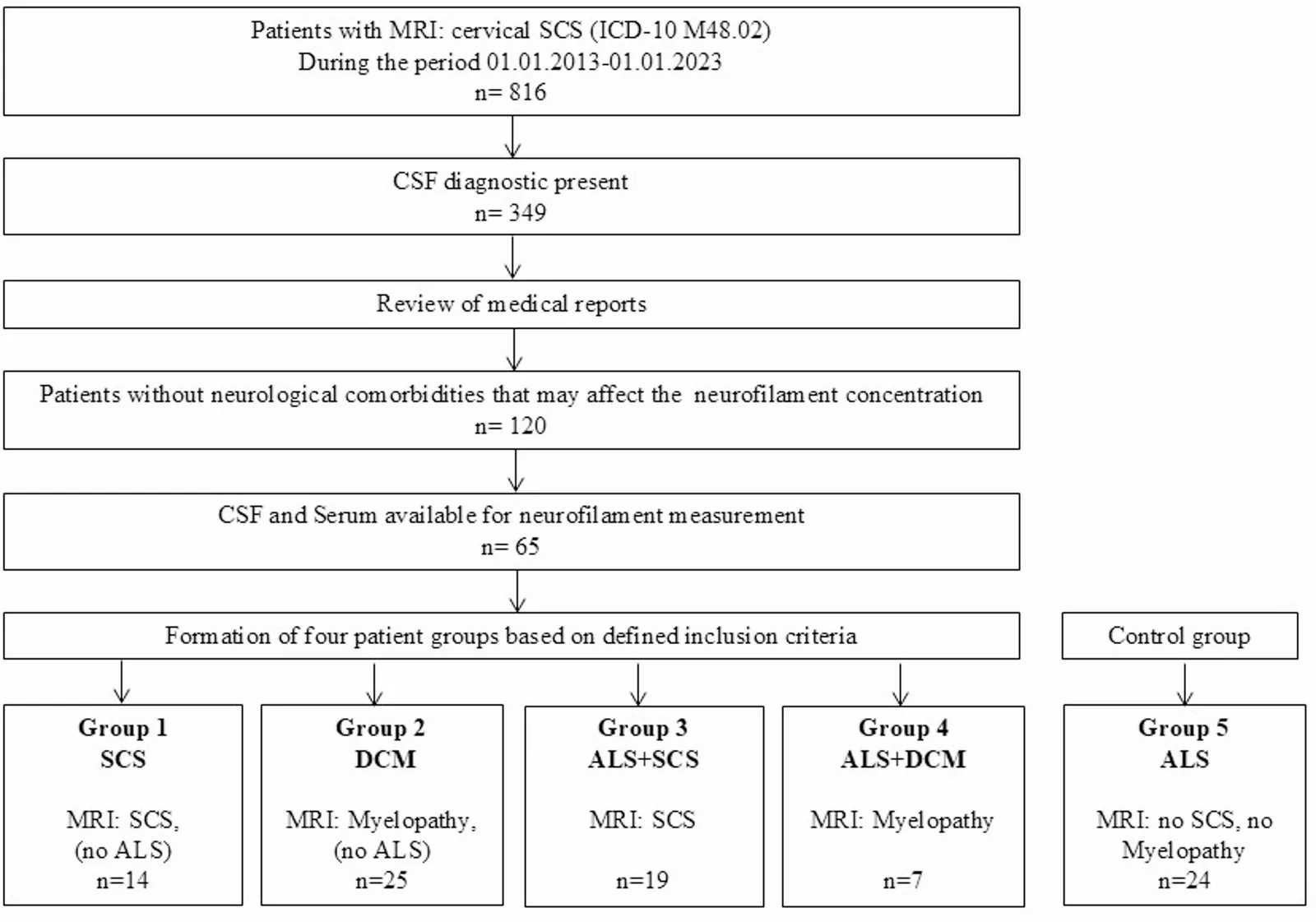
Patient inclusion flowchart. n refers to the corresponding number of patients who could be included after the evaluation of the respective criterion

Data from the Department of Neurology, University Hospital Ulm, Germany were available for this study. The study had been approved by the Ethics Committee of the University of Ulm (reference number 113/24) and was conducted in accordance with the Declaration of Helsinki. All patients gave written informed consent for the use of remaining samples for research. Patients treated in the Department during the period from 01/Jan/2013 to 01/Jan/2023 were considered for this study. Those who were diagnosed with cervical spinal canal stenosis (ICD-10 M48.02) on MRI and who had undergone lumbar puncture with CSF diagnostics during their stay were eligible for inclusion (Fig. 1).

If other neurological diseases were diagnosed that could influence neurofilament concentrations, the patient was excluded. These disorders include inflammatory CNS diseases, acute CNS injuries such as stroke, infectious CNS diseases and CNS tumours or metastases.

The inclusion criteria differed between the groups and were based on the clinical presentation and MRI findings. In order for patients to be included in group 1 to 4, SCS in the cervical region had to be described in MRI Imaging. Patients assigned to the DCM group were required to present with clinical signs consistent with degenerative cervical myelopathy and myelopathy confirmed by imaging. Therefore, for inclusion in group 2 and 4, a myelopathy signal had to be present in T2-weighted MRI. ALS diagnosis was established according to the revised El Escorial criteria (Brooks et al. 2000) after comprehensive diagnostic evaluation to exclude differential diagnoses in accordance with established diagnostic guidelines. Progression rate (ΔFS) was calculated as: (ΔFS)=(48-ALSFRS-R at diagnosis or initial assessment/disease duration (in month) (Alves et al. 2025; Kimura et al. 2006). Demographic data of the ALS patients is provided in Table 1. The Age at onset for the ALS groups varied between 63.5 (group 4) and 64.6 (group 3) (Table 1). Age at lumbar puncture (LP) was between 65.3 (group 4) and 66.1 (group 3) (Table 1).

The first group included patients with isolated spinal canal stenosis (group 1, *n* = 14, male:9, female:5), the second group included patients with degenerative cervical myelopathy (group 2, *n* = 25, male:18, female:7), the third group included patients with spinal canal stenosis and ALS (group 3, *n* = 19, male:10, female:9), and the fourth group included patients with cervical myelopathy and ALS (group 4, *n* = 7, male:6, female:1). The fifth group served as controls and included patients with ALS only who did not show evidence of spinal canal stenosis or myelopathy in imaging (group 5, *n* = 24, male:13, female:11). This group was blinded and matched to group 2 in terms of age, with a maximum age difference of five years accepted.

One additional inclusion criterion was the availability of serum and CSF samples in the Biobank of the RKU (University Hospital Ulm). With informed patient consent, serum and CSF samples are stored in The Biobank of the RKU for research purposes under standardized conditions at -80 °C. Neurofilaments were measured using the Ella™ Automated Immunoassay System (Bio-techne, Minneapolis, USA). This is an automated system based on the principle of immunoassays, which can quantify the concentration of neurofilaments in CSF and serum (software: Simple Plex Runner Version 4.1.0.22). Recombinant and in-house controls were determined for each measurement. The total number of patients (n) may vary between groups, as not every patient had a CSF or serum sample available foranalysis. 

The albumin quotient is age-dependent and can be calculated using the Reiber formula. The upper reference limit was calculated using the age-adjusted formula: QAlb = [4+( Age in years/15)] x10^− 3^ (Reiber et al. 2001).

Z-scores allow for age-adjusted interpretation of NfL concentrations in CSF and serum. These reference values were developed as part of a previous study conducted by our group (Halbgebauer et al. 2024). Using these Z-score reference values, the measured neurofilament levels of the five groups were categorized. Z-scores above 1 were classified as mildly elevated, above 1,5 as elevated and above 2 as strongly elevated. Statistical analysis was performed using IBM SPSS Statistics Version 29.0.1.0 (IBM SPSS Statistics, Armonk, New York, USA). After testing for normal distribution, the results first underwent a descriptive analysis. The Kruskal-Wallis test was used for non-normally distributed, continuous data (neurofilaments, QAlb, albumin in CSF and serum, and age). This was followed by a post-hoc analysis with the Dunn-Bonferroni test for pairwise comparisons. The chi-square test was used for categorical variables such as gender and neurological diagnoses. 

## Results

**Fig. 2:**
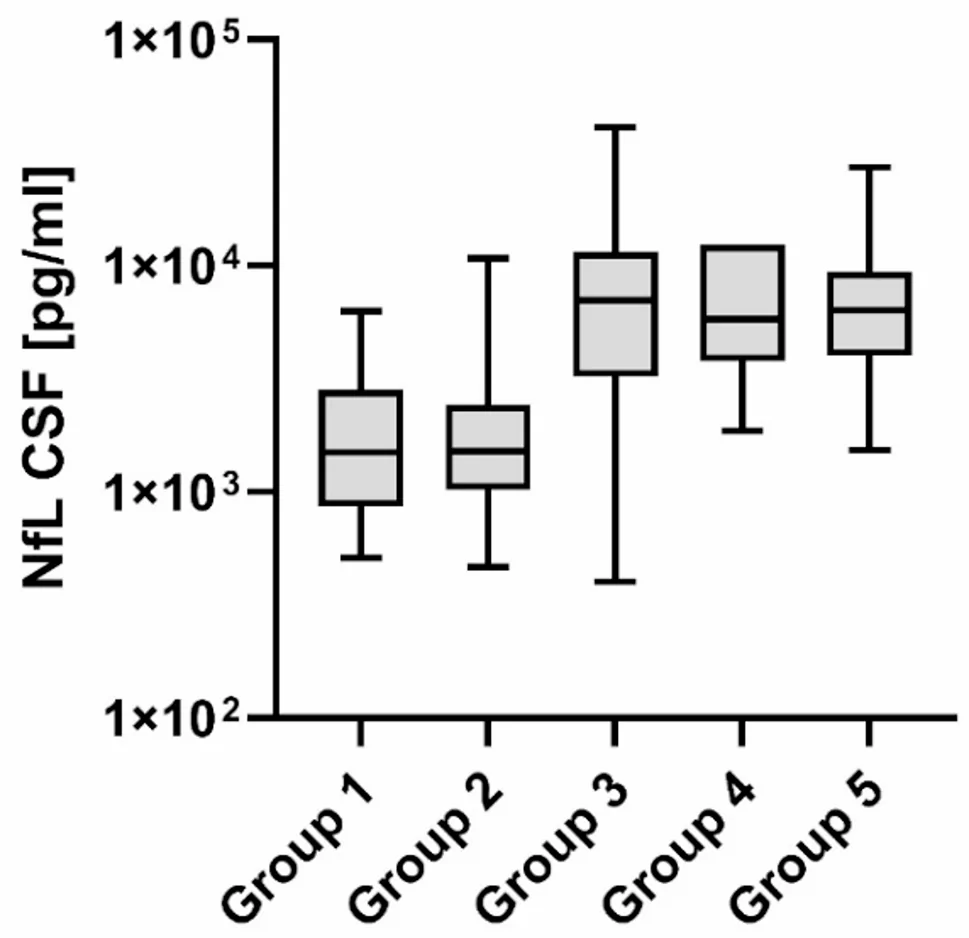
Concentrations of NfL in CSF [pg/ml]

**Fig. 3:**
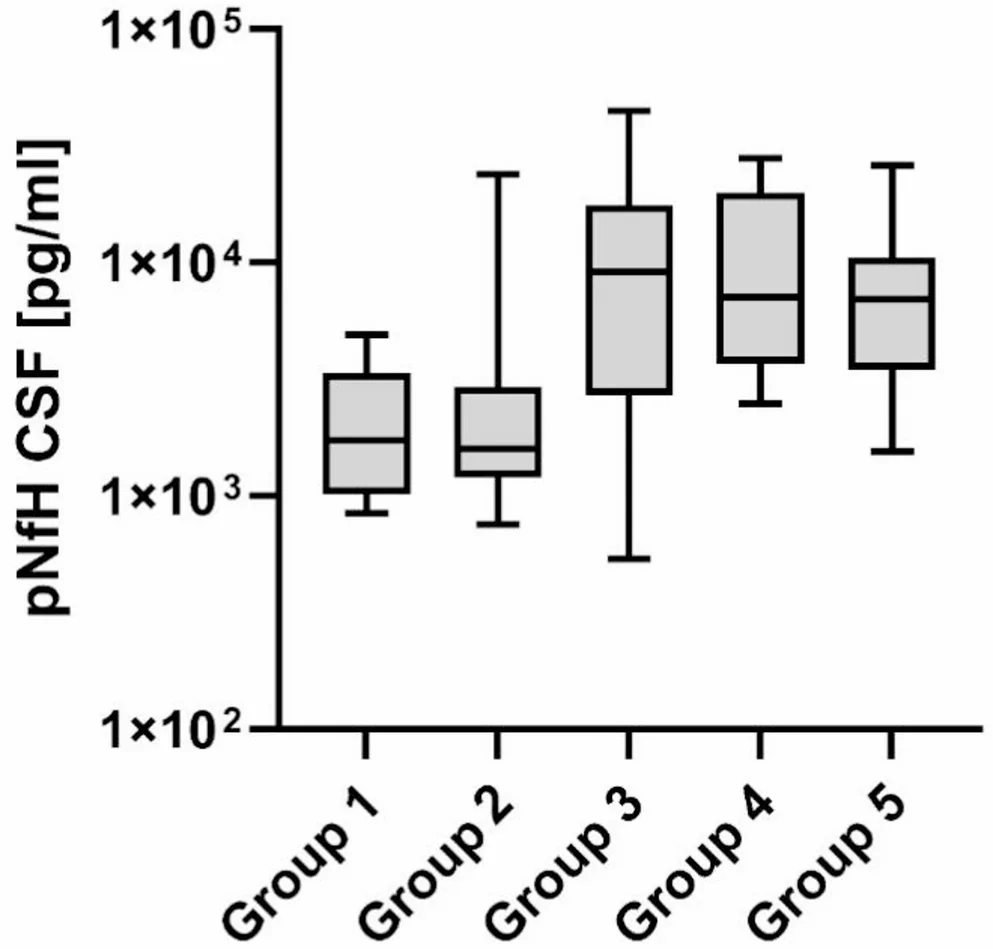
Concentrations of pNfH in CSF [pg/ml]

**Fig. 4:**
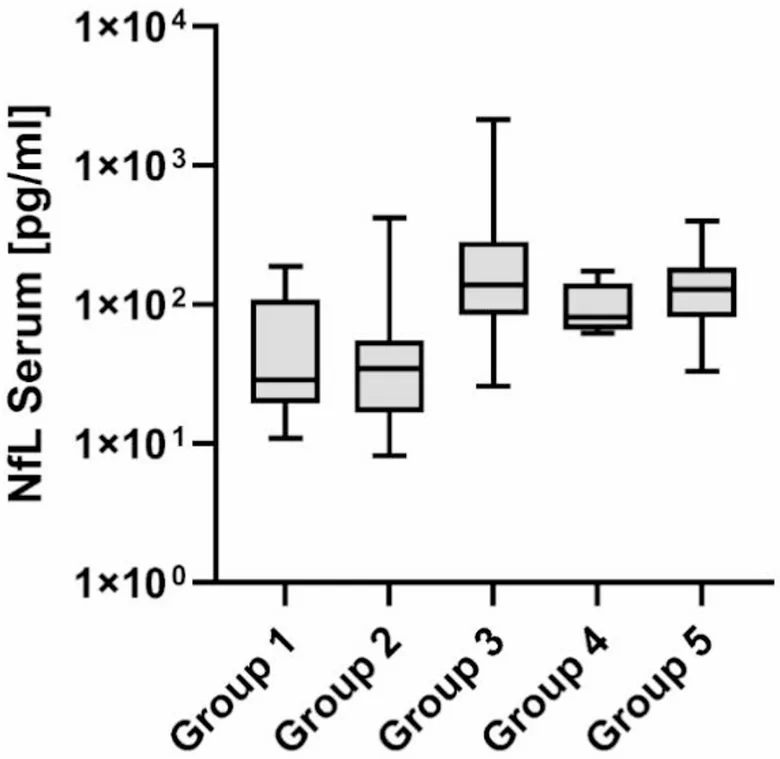
Concentrations of NfL in Serum [pg/ml]

**Fig. 5:**
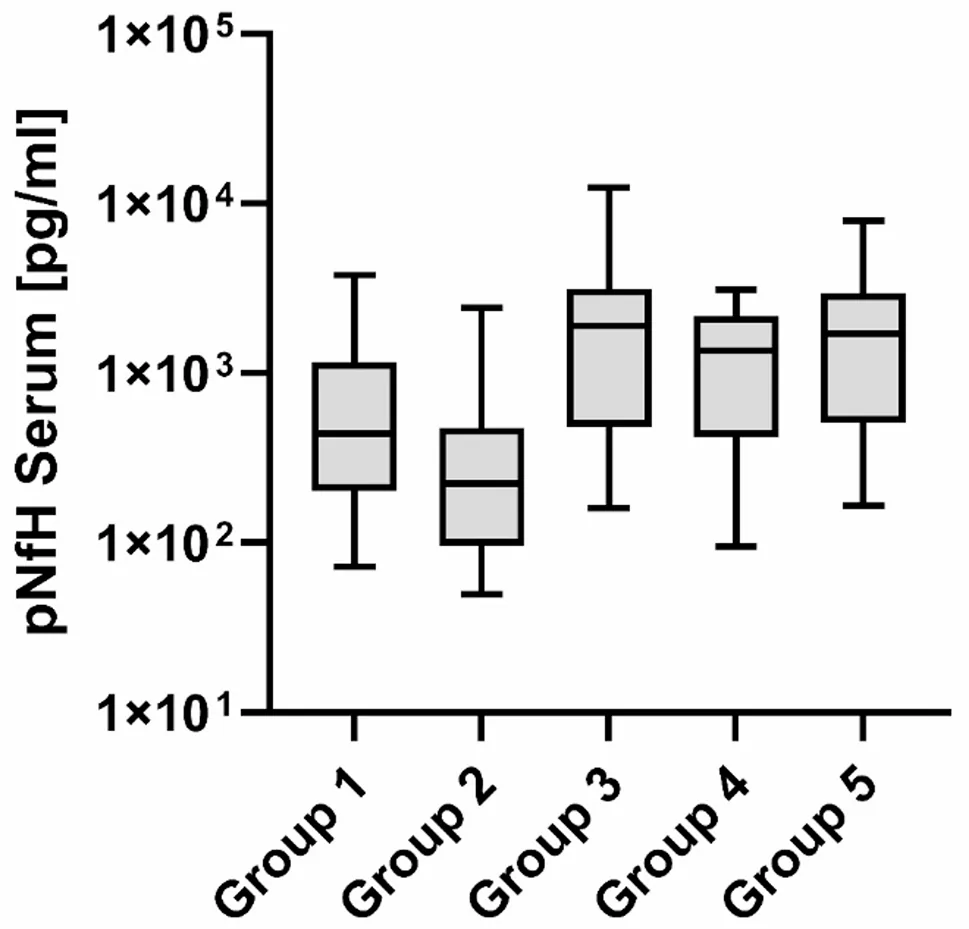
Concentrations of pNfH in serum [pg/ml]

**Fig. 6:**
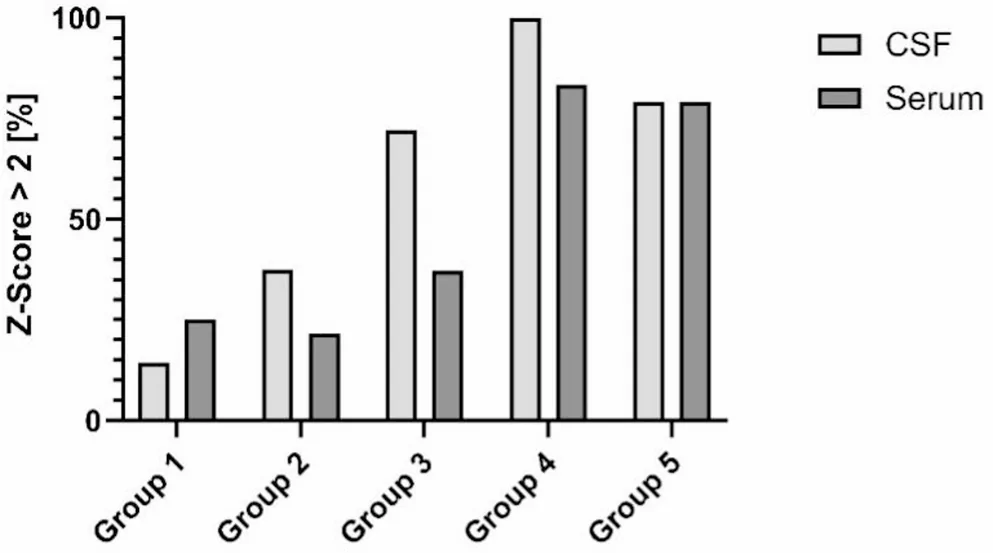
Proportion of strongly elevated Z-Scores (> 2) in CSF and Serum

The age of the patients was similar in all groups (*p* > 0.05), with a median between 62 and 69 years. Comparison of neurofilament concentrations between patients with isolated spinal canal stenosis (group 1) and patients with cervical myelopathy (group 2) revealed no significant difference (Table 3). The Z-scores of groups 1 and 2 showed a similar distribution. In serum, the NfL scores of a high proportion of patients were within the normal range (group 1: 50% and group 2: 69.6%) (Table 4). In contrast, the results of the Z-scores in the CSF were often elevated. Group 1, 50% had elevated neurofilaments and 14.3% had strongly elevated NfL concentrations (Table 4). 37.5% in group 2 showed strongly elevated neurofilaments (Table 4). Pairwise comparisons of group 1 and group 2 with the individual ALS groups revealed significant differences (*p* < 0.05) (Table 3). This result was consistent for NfL in CSF and serum as well as for pNfH in CSF except for comparisons with group 4. For pNfH in serum, a significant difference was only found when comparing group 2 with group 3 and group 5, respectively (Table 3). The Z-scores are consistent with these results. A lower percentage (37.5%) of the patients in group 2 had strongly elevated NfL levels in CSF in comparison to 79.2% in Group 5, while in serum, 21.7% had strongly elevated NfL levels compared to 79.2% in Group 5 (Table 4). Compared to groups 1 and 2, the groups with ALS patients (groups 3, 4 and 5) showed a higher proportion of elevated NfL Z-scores, as well as measured neurofilament concentrations, both in CSF and in serum (Table 2 and Figs. 2, 3, 4 and 5). The QAlb was more frequently abnormal in group 1 and 2 (42.9% and 40%), compared to the control group 5 (25%). The highest proportion of patients with a blood-CSF barrier dysfunction, indicated by an elevated QAlb, was in group 4 (71.4%), where the ALS diagnosis as well as a myelopathy was present (Table 5). No consistent co-occurrence of pathological QAlb elevation and increased neurofilament concentrations was observed (Table 6). The occurrence of elevated NfL concentrations in CSF and serum was comparable in patients with elevated QAlb and those with normal QAlb (Table 6). Clinical findings at lumbar puncture differed between group 2 (DCM) and ALS (group 5), with atrophy and paresis being among the most prevalent finding in both cohorts (Table 7). Spasticity and fine motor and coordination dysfunction were more common in the DCM group compared to the ALS group (Table 7) (Fig. 6).

**Fig. 7:**
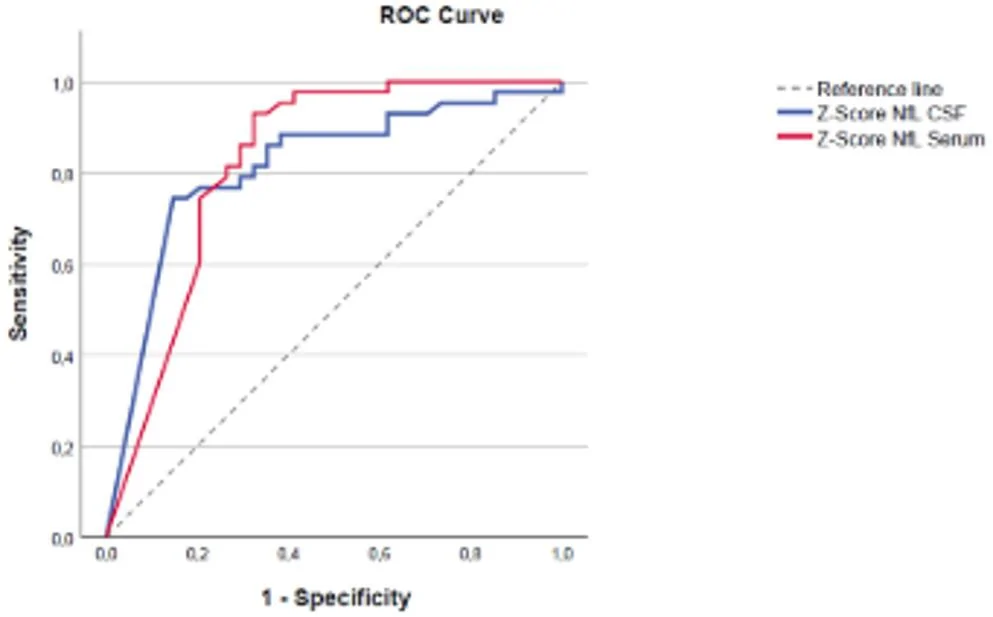
ROC curve

ROC curve analysis was performed to evaluate the diagnostic performance of age-adjusted serum and CSF NfL Z-scores for differentiating ALS from non-ALS patients (SCS and DCM). Both biomarkers demonstrated good diagnostic accuracy (Fig. 7). The CSF NfL z-score showed an area under the curve (AUC) of 0.813 (95% CI 0.712–0.913), whereas the serum NfL Z-score showed an AUC of 0.823 (95% CI 0.721–0.926) (Fig. 7).

**Table 1:** Demographic data ALS patients

	Group 3 ALS + SCS (*n* = 19)	Group 4 ALS + DCM (*n* = 7)	Group 5 ALS (*n* = 24)	All (*n* = 50)
Sex male/female, *n* (%)	10/9 (52.6%/47.4%)	6/1 (85.7%/14.3%)	13/11 (54.2%/45.8%)	29/21 (58%, 42%)
Age at onset, Mean (SD)	64.6 (10.2)	63.5 (10.2)	63.8 (11.4)	64 (10.6)
Age at LP, Mean (SD)	66.1 (10.5)	65.3 (10.6)	65.0 (11.0)	65.4 (10.5)
BMI, Mean (SD)	26.06 (4.69)	25.32 (2.5)	25.81 (5.2)	25.8 (4.54)
Positive family history, n (%)	2 (10.5%)	2 (28.6%)	4 (16.7%)	8 (16%)
Onset site spinal, bulbar, thoracic n (%)	11,4,1 (57.9%, 21.1%, 5.3%)	7,0,0 (100%, 0%, 0%)	15,8,0 (62.5%, 33.3%, 0%)	33,12,1 (66%, 24%, 2%)
Progression rate, mean (SD)	1.06 (0.8)	0.64 (0.3)	0.81 (0.7)	0.871 (0.7)

**Table 2:** Neurofilament concentrations

Group	MD	IQR	M	SD	Min	Max	Median Z-score	Fold-change compared to Group 2 (DCM)
*NfL in CSF [pg/ml]*								
1 SCS (*n* = 14)	1491.5	1975	2093.4	1609.7	510	6303	1.64	0.99
2 DCM (*n* = 24)	1513.5	1416	2321.0	2402.2	467	10820	1.52	1.00
3 ALS + SCS (*n* = 18)	7050.0	8265	9200.2	9442.2	402	40793	2.5	4.66
4 ALS + DCM (*n* = 7)	5815.0	8592	7289.4	4131.6	1867	12430	2.5	3.84
5 ALS (*n* = 24)	6347.5	5439	8323.3	6868.4	1525	27336	2.5	4.19
*pNfH in CSF [pg/ml]*								
1 SCS (*n* = 14)	1723.0	2359	2149.9	1252.3	837	4904		1.08
2 DCM (*n* = 24)	1590.5	1717	3479.9	5094.7	755	23952		1.00
3 ALS + SCS (*n* = 17)	9127.0	14923	13215.9	13208.5	534	44,441		5.74
4 ALS + DCM (*n* = 7)	7108.0	16257	10829.0	9524.3	2486	27933		4.47
5 ALS (*n* = 24)	6967.5	7004	7656.6	5745.9	1549	26088		4.38
*NfL in Serum [pg/ml]*								
1 SCS (*n* = 12)	28.7	88.8	66.1	65.1	10.9	188.0	0.87	0.83
2 DCM (*n* = 23)	34.6	39.5	74.0	104.8	8.1	419.0	0.44	1.00
3 ALS + SCS (*n* = 15)	137.5	200.1	321.5	544.1	25.8	2129.0	2.5	3.97
4 ALS + DCM (*n* = 6)	81.0	77.5	99.7	44.4	62.6	174.0	2.33	2.34
5 ALS (*n* = 24)	128.0	103.9	148.5	88.6	32.9	398.0	2.5	3.7
*pNfH in Serum [pg/ml]*								
1 SCS (*n* = 12)	442.0	958.5	851.2	1091.4	72.0	3797.0		1.98
2 DCM (*n* = 22)	223.5	380.7	457.4	629.8	49.9	2435.0		1.00
3 ALS + SCS (*n* = 15)	1898.0	2671.0	2876.5	3574.0	160.0	12365.0		8.49
4 ALS + DCM (*n* = 6)	1357.5	1734.0	1379.5	1068.9	95.2	3092.0		6.07
5 ALS (*n* = 24)	1714.5	2444.5	2162.0	1885.9	165.0	7930.0		7.67

For each group, the median (MD), interquartile range (IQR), mean (M), standard deviation (SD), minimum (Min), and maximum (Max) are reported

*n* Total number of cases, Fold change is in comparison to the Median of group 2, *SCS* spinal canal stenosis, *DCM* degenerative cervical myelopathy, *ALS* amyotrophic lateral sclerosis

**Table 3:** Results of the Kruskal Wallis test comparing the concentrations of NfL and pNfH in CSF and serum

Comparison of groups	NfL CSF *p*-value	pNfH CSF *p*-value	NfL serum *p*-value	pNfH serum *p*-value
1–2	1.00	1.00	1.00	1.00
1–3	0.008*	0.005*	0.039*	0.356
1–4	0.048*	0.03*	1.00	1.00
1–5	0.001*	0.005*	0.027*	0.165
2–3	0.002*	0.003*	0.003*	0.002*
2–4	0.031*	0.033*	0.955	0.394
2–5	< 0.001*	0.003*	0.001*	< 0.001*
3–4	1.00	1.00	1.00	1.00
3–5	1.00	1.00	1.00	1.00
4–5	1.00	1.00	1.00	1.00

For each variable, pairwise group comparisons were performed. The adjusted p-values obtained from the Kruskal-Wallis test with subsequent post-hoc analysis using Bonferroni correction are reported. Significant p-values (*p* < 0,05) are marked with an asterisk (*)

**Table 4:** Z-scores of CSF NfL

Z-Score: NfL	Group 1 SCS		Group 2 DCM		Group 3 ALS + SCS		Group 4 ALS + DCM		Group 5 ALS	
	**CSF** (*n* = 14)	**Serum** (*n* = 12)	**CSF** (*n* = 24)	**Serum** (*n* = 23)	**CSF** (*n* = 18)	**Serum** (*n* = 15)	**CSF** (*n* = 7)	**Serum** (*n* = 6)	CSF (*n* = 24)	Serum (*n* = 24)
Normal < 1	4 (28.6%)	6 (50%)	5 (20.8%)	16 (69.6%)	1 (5.6%)	1 (6.7%)	0 (0%)	0 (0%)	1 (4.2%)	1 (4.2%)
Lightly elevated [1;1.5]	1 (7,1%)	2 (16.7%)	7 (29.2%)	0 (0%)	2 (11.1%)	2 (13.3%)	0 (0%)	1 (16.7%)	1 (4.2%)	1 (4.2%)
Elevated ]1.5; 2]	7 (50%)	1 (8.3%)	3 (12.5%)	2 (8.7%)	2 (11.1%)	1 (6.7%)	0 (0%)	0 (0%)	3 (12.5%)	3 (12.5%)
Strongly elevated > 2	2 (14.3%)	3 (25%)	9 (37.5%)	5 (21.7%)	13 (72.2%)	11 (73.3%)	7 (100%)	5 (83.3%)	19 (79.21%)	19 (79.2%)

Shown is the distribution of the Z-score results. Given are the percentages (%) of the total group size (n)

**Table 5:** Blood-CSF barrier dysfunction, indicated by QAlb above the reference value

Group	QAlb above the reference value
1 SCS (*n* = 14)	6 (42.9%)
2 DCM (*n* = 25)	10 (40%)
3 ALS + SCS (*n* = 19)	5 (26.3%)
4 ALS + DCM (*n* = 7)	5 (71.4%)
5 ALS (*n* = 24)	6 (25%)

The table shows the number of patients with an elevated albumin quotient (QAlb) in each group. The percentage (%) relating to the total number of patients (n) is indicated

**Table 6:** Association between QAlb and elevated NfL concentrations in serum and CSF

	QAlb elevated		QAlb normal	
	Serum NfL elevated	CSF NfL elevated	Serum NfL elevated	CSF NfL elevated
1 SCS	33.3%	66.7%	42.9%	62.5%
2 DCM	12.5%	60%	40%	42.9%
3 ALS + SCS	100%	100%	75%	76.9%
4 ALS + DCM	80%	100%	100%	100%
5 ALS	100%	100%	88.9%	88.9%

Elevated NfL concentrations were defined by the age-adjusted Z-scores above 1.5. Percentages (%) refer to the total number of patients with either elevated or normal QAlb within the respective group

**Table 7:** Clinical findings at the time of lumbar puncture

	Group 2 DCM (*n* = 25)	Group 5 ALS (*n* = 24)
Atrophy	18 (72%)	22 (91.7%)
Fasciculations	9 (36%)	16 (66.7%)
Paresis	15 (60%)	24 (100%)
Spasticity	8 (32%)	3 (12.5%)
Positive Babinski sign	6 (24%)	8 (33.3%)
Fine motor and coordination dysfunction	12 (48%)	8 (33.3%)
Gait disturbance	16 (64%)	13 (54.2%)
Bladder dysfunction	4 (16%)	3 (12.5%)

## Discussion

Comparison of NF concentrations between patients with isolated SCS (Group 1) and DCM (Group 2) revealed no significant differences. In contrast, patients with ALS (Groups 3, 4,5) showed significantly higher NfL and pNfH levels compared to group 1 and 2. The results of the Z-scores are consistent with these findings.

The non-significant difference in NF concentrations between groups 1 and 2 suggests that a similar degree of axonal damage occurs in both spinal canal stenosis and DCM. These findings are partially consistent with the results of other studies in which elevated neurofilament concentrations were described primarily in patients with acute clinical worsening (Kato et al. 2015; Takahashi et al. 2014). The observed discrepancy between studies that report increased neurofilaments in DCM and studies like the current one, not showing an increase, may therefore be explained by differences in disease duration, myelopathy severity, acute disease courses, and individual pathophysiology.

It is reasonable to assume that in acute deterioration of neurological deficits, active neuronal damage occurs, leading to the release of neurofilaments into the CSF. In contrast, in chronic disease, structural remodelling processes may have already taken place, resulting in lower neurofilament release. The onset and clinical course of the myelopathy could not be taken into account in this study, which limits our interpretation of the neurofilament concentrations.

These results are consistent with the findings of other studies showing a significant increase in neurofilaments in ALS patients (Steinacker et al. 2016). Therefore, it can be assumed that there is a higher degree of axonal damage in ALS compared to degenerative compression disorders of the cervical spine such as SCS and DCM. It should be noted that low neurofilament concentrations do not rule out an ALS diagnosis, but are preferentially observed in more benign variants (Gille et al. 2019). In such cases, differential diagnosis is challenging and requires a high degree of diagnostic accuracy. It should also be noted that NF levels are already elevated early in the disease course of ALS (Feneberg et al. 2018; Gille et al. 2019; Lu et al. 2015). Since pNfH and NfL are expressed in the CNS as well as in PNS and are not specific to a particular disease, their diagnostic relevance can only be determined in the clinical context (Khalil et al. 2024; Thelin et al. 2017). Also elevated NF levels may reflect neuroaxonal damage caused by various, sometimes overlapping pathologies (Khalil et al. 2024). Therefore, comorbidities should be taken into account, as they may also contribute to increased NF levels (Khalil et al. 2024). An advantage of NF is the strong correlation in serum and CSF, enabling longitudinal monitoring of neuronal injury (Halbgebauer et al. 2022; Khalil et al. 2024).

Although the present findings support the diagnostic value of neurofilament biomarkers, the absence of standardized measures of disease severity precluded further analyses of their relationship with clinical disease burden. Consequently, the diagnostic performance observed in this cohort should be interpreted with caution, as important information such as disease duration, progression rate and for example site of onset were not included in the study. This study primarily focused on neurofilament biomarkers. However, biomarkers capable of distinguishing between central and peripheral neuronal damage, such as peripherin (PRPH) (Bombaci et al. 2025), may further improve the differential diagnosis of ALS. Future studies of other interesting biomarkers should evaluate their diagnostic performance and potential complementary value alongside neurofilament biomarkers.

The results of this study showed that barrier dysfunction was more common in myelopathy patients than in the ALS group. Pathophysiologically, this result is understandable if one assumes that mechanical compression leads to structural and functional damage to the integrity of the blood/CSF barrier (Kim et al. 2023). Studies have shown that barrier dysfunction occurs particularly in cases of pronounced compression (Blume et al. 2020). It remains unclear whether mechanical compression is an independent factor leading to barrier dysfunction or whether its effects occur secondarily to compression-induced ischemia (Kim et al. 2023).

To date, only few studies have investigated changes in the albumin quotient in ALS patients: Klose et al. 2024 showed that ALS patients did not show significantly different QAlb values from control cohorts, but the albumin quotient correlated with disease severity and survival of ALS patients (Klose et al. 2024). It is currently unclear whether barrier dysfunction associated with ALS contributes to the loss of motor neurons or occurs as a consequence of motor neuron dysfunction (Ouali Alami et al. 2020). The proportion of age-adjusted pathologically elevated albumin quotient values was comparable in the group with only SCS (group 1) and in the group with cervical myelopathy (group 2). The result could emphasize the common pathophysiological basis of SCS and DCM. The exact pathophysiological role of barrier dysfunction in the course of the disease is a research question for further future studies. The extent to which elevated QAlb values are associated with a higher severity of compression is beyond the scope of the present study. The QAlb cannot be used for differential diagnosis currently, but in combination with other biomarkers, it might be useful for assessing disease progression and prognosis, particularly in ALS.

### We see the following limitations of our study

Since cervical myelopathy is often accompanied by multimorbidity, it was not our aim to recruit large patient groups, but rather to enroll only clearly defined patients through careful preselection and review of the medical records. This resulted in a limited number of patients in each cohort, which were however clearly defined clinically. The number of patients included in each group should be considered when looking at statements about significant differences. The small sample size may have limited the statistical power to detect differences between groups. Therefore, some pairwise comparisons should be interpreted with caution, as a Type II error cannot be excluded.

The NF concentrations can be influenced by BMI and renal function (Witzel 2024), but these parameters were not taken into account when interpreting the NF concentrations. In order to assess the progression of DCM based on neurofilament concentrations, the progression of degenerative changes in the cervical spine should be taken into account, but longitudinal data were not available for DCM Patients. Clinically relevant information, such as disease progression rate and disease severity, were only available for ALS patients. Even though the data were collected, they were not included in the analysis of NF concentrations. 

## Conclusion

It was shown that neurofilament concentrations in cervical SCS, with and without myelopathy signal in MRI, differed significantly from ALS. Neurofilaments can therefore support the diagnostic differentiation from DCM but should always be interpreted in the context of clinical findings and imaging results. The results support the role of neurofilaments, including the use of Z-scores, as increasingly important biomarkers in the differential diagnosis of neurodegenerative diseases.

## Data Availability

All data are available from the corresponding author upon reasonable request.
